# iCub-HRI: A Software Framework for Complex Human–Robot Interaction Scenarios on the iCub Humanoid Robot

**DOI:** 10.3389/frobt.2018.00022

**Published:** 2018-03-12

**Authors:** Tobias Fischer, Jordi-Ysard Puigbò, Daniel Camilleri, Phuong D. H. Nguyen, Clément Moulin-Frier, Stéphane Lallée, Giorgio Metta, Tony J. Prescott, Yiannis Demiris, Paul F. M. J. Verschure

**Affiliations:** ^1^Personal Robotics Laboratory, Electrical and Electronic Engineering Department, Imperial College, London, United Kingdom; ^2^Synthetic Perceptive Emotive and Cognitive Systems Group (SPECS), Universitat Pompeu Fabra, Barcelona, Spain; ^3^Institute for Bioengineering of Catalonia (IBEC), The Barcelona Institute of Science and Technology, Barcelona, Spain; ^4^Department of Computer Science, University of Sheffield, Sheffield, United Kingdom; ^5^iCub Facility, Istituto Italiano di Tecnologia, Genova, Italy; ^6^ICREA-Institució Catalana de Recerca i Estudis Avançats, Barcelona, Spain

**Keywords:** robotics, iCub humanoid, human–robot interaction, YARP, software architecture, code:C++, code:Python, code:Java

## Abstract

Generating complex, human-like behavior in a humanoid robot like the iCub requires the integration of a wide range of open source components and a scalable cognitive architecture. Hence, we present the iCub-HRI library which provides convenience wrappers for components related to perception (object recognition, agent tracking, speech recognition, and touch detection), object manipulation (basic and complex motor actions), and social interaction (speech synthesis and joint attention) exposed as a C++ library with bindings for Java (allowing to use iCub-HRI within Matlab) and Python. In addition to previously integrated components, the library allows for simple extension to new components and rapid prototyping by adapting to changes in interfaces between components. We also provide a set of modules which make use of the library, such as a high-level knowledge acquisition module and an action recognition module. The proposed architecture has been successfully employed for a complex human–robot interaction scenario involving the acquisition of language capabilities, execution of goal-oriented behavior and expression of a verbal narrative of the robot’s experience in the world. Accompanying this paper is a tutorial which allows a subset of this interaction to be reproduced. The architecture is aimed at researchers familiarizing themselves with the iCub ecosystem, as well as expert users, and we expect the library to be widely used in the iCub community.

## 1 Introduction and Background

The iCub is an advanced humanoid robot, which is equipped with multiple sensors: encoders in all its 53 joints, force/torque sensors, tactile sensors integrated in the artificial skin, and eye cameras (Metta et al., 2010). They allow for a coherent understanding of body configuration, motor capabilities, and the environment as well as an ability to show facial expressions, which makes it an ideal platform for studies of human–robot interaction and cognition.

The research community around the iCub humanoid robot is very active, with a large number of papers published every year. The source code leading to these publications is often made available to the public, which allows for the replication of the results and use of the code as a starting platform to tackle new research questions. However, despite YARP (Fitzpatrick et al., 2006) being typically used as the underlying middleware in these works, it remains challenging to combine these efforts in a coherent manner.

Here, we present iCub-HRI, which integrates several components for perception, object manipulation, and social interaction using two parts: (1) The iCub-HRI library, which facilitates the use of the aforementioned components by providing easy to use classes with suitable default parameters (called *Subsystems*) and a shared knowledge database as means to represent knowledge which is employed across all components. (2) Modules which supply the shared knowledge database with input, as well as some modules tailored for human–robot interaction scenarios.

### 1.1 Background and Related Works

iCub-HRI has its origins in the Experimental Functional Android Assistant (EFAA) project,^1^ where most of the library was developed and employed in several works (e.g., Lallée et al. (2013, 2015), Petit et al. (2013), and Lallée and Verschure (2015)). EFAA targeted the development of a cognitive architecture to realize effective and psychologically plausible human–robot dyadic interaction. The code was then extended and matured further in the What You Say Is What You Did (WYSIWYD) project,^2^ and more papers based on iCub-HRI were published (e.g., Fischer and Demiris (2016), Martinez-Hernandez et al. (2016), Petit et al. (2016), Puigbò et al. (2016), and Moulin-Frier et al. (2017)). WYSIWYD aimed at realizing robot human level language capabilities by augmenting this cognitive architecture with mechanisms for language acquisition, composition, and expression. The cognitive architecture in both projects is based on and elaborates the Distributed Adaptive Control theory of mind and brain (DAC, see for reviews Verschure (2012, 2016) and Section 4.3).

While reviewing robotics middlewares is out of the scope for this paper (we refer to Elkady and Sobh (2012) for an overview), we briefly introduce several other proposals detailing software frameworks for various robotics platforms. Natale et al. (2016) summarize recent developments of the iCub’s software architecture, including the compatibility with the Robot Operating System (ROS) and the introduction of a new testing framework. They find that ROS is being adopted rapidly by more and more robot developers, and indeed, there are several papers introducing human–robot interaction-related frameworks based on ROS. For example, Jang et al. (2015) propose a ROS-based framework where modules concerned with low-level control and service logic are separated from modules concerned with social behaviors. Lane et al. (2012) present a bundle of ROS modules which allows the extension of existing projects for speech recognition, natural language understanding, and basic gesture recognition as well as gaze tracking. A toolkit which allows the evaluation of human–robot interactions in virtual reality environments and subsequent deployment on a real robot was presented by Krupke et al. (2017). The robot behavior toolkit (Huang and Mutlu, 2012) includes a ROS module which is based on the findings within the social sciences. While the authors conducted a large-scale study with humans, the evaluation was based on simulated sensor data. Finally, Sarabia et al. (2011) present a framework allowing to perceive the actions and intentions of humans, and show its application in a social context where a robot imitates the dance movements of a human.

### 1.2 Design Principles

Here, we devise a set of guidelines and design principles which were adopted when coding the framework.

- *Adaptability and ease of use*: the framework should be easy to adapt by the community. Individual parts of the framework should only depend on other parts if necessary, and substituting components should be easy. Furthermore, all libraries and modules should be properly documented.
- *Provision of overall framework*: related to the previous goal, our aim is to provide an overall framework which can work “out of the box.” Hence, our framework contains modules related to perception, action execution, and social interaction.
- *Extendibility*: it should be easy to extend the framework with new modules. Rather than tailoring existing modules to work with the iCub-HRI framework, it should be possible to write wrapper code for the integration.
- *Shared, centralized knowledge representation*: each module should have access to the same knowledge database, and the contained knowledge should follow a standardized format. Within iCub-HRI, we call this knowledge database the *working memory*, and the contents are *Entities* or derivatives thereof. The working memory is the default means of communicating among modules.
- *Open software*: the code is released open source and made publicly available. All dependencies must be available as open source software too.

## 2 The iCub-HRI Library

Due to the support of distributed computation within the YARP middleware, there are typically many modules running simultaneously when conducting research on the iCub. Typically, data are exchanged using YARP’s *Bottle* container, which can encapsulate data of arbitrary length and varying type. While this allows a high degree of flexibility, these containers are error prone due to the requirement of parsing the messages dynamically. This makes verification of compatibility and versioning when used across a large number of modules hard (Natale et al., 2016). Thus, within the iCub-HRI library, we introduce fixed data representations for knowledge (fully compatible with the *Bottle* container), similar to those used in ROS messages (Quigley et al., 2009) and the Interface Description Language (IDL) in YARP (Fitzpatrick et al., 2014). Contrary to ROS messages and IDLs, the same representations are used across all components of the iCub-HRI library. The representations and their exchange which is orchestrated by a working memory are detailed in Section 2.1.

The communication protocol with external modules is described within *Subsystems*. Each subsystem connects to a host (i.e., external module) and abstracts away the communication internals, as described in Section 2.2. Finally, the *icubClient* class is designed with additional convenience for end users in mind such that all subsystems and other higher level methods are available from within a single class.

### 2.1 Knowledge Representation and Exchange

The basic representation type is an *Entity*, which is specified by an *ID* and an associated *name*. The *ID* is used when storing and retrieving the entity from the working memory. Several entities can be linked together by the means of a *Relation*, for example, the human “Paul” (subject) “holds” (verb) “duck” (object). For further details on relations, we refer to Lallée and Verschure (2015).

Other knowledge representations inherit the basic properties and methods of *Entity* and extend them further. The *Object* class has additional properties representing the pose, size, presence, and saliency of an object (see Section 3.1 for details how these properties are acquired). The *Agent* class represents a human partner, which additionally to all properties of an *Object* also stores the positions of all body parts and a list of beliefs. Another commonly used representation is that of a *Bodypart*, which represents a part of the robot’s body. A *Bodypart* also inherits all attributes of an *Object*, and additionally contains the related joint number, tactile patch identifier, and corresponding body part of the human. Zambelli et al. (2016) have used these representations to anchor self-learned representations to those of a human interacting with the robot.

These representations must be shared across different modules (for example, between perceptual modules and the more abstract reactive layer as described later in this section), and we designed the *OPCClient* class to automate the exchange of representations with the working memory of the iCub ecosystem (Objects Properties Collector; OPC) (Lallée and Verschure, 2015). The OPC is an ontology-based knowledge representation system which is grounded on the need of humans and other social animals to interact in a physical, multi-agent world (see Lallée and Verschure (2015)). In this direction, the role of such knowledge representation should be to structure and distribute information to different modules in an asynchronous (on-demand) and centralized way. The design is inspired by the *repository pattern* known from software engineering (Evans, 2004), and its usage is very similar to the centralised version control software Apache Subversion (known as SVN).^3^ For storage and retrieval, the *OPCClient* provides methods such as “checkout” to poll representations from the shared memory, “update” to update existing representations, and “commit” to overwrite representations in the memory with the local version of the module. Altogether, this implementation provides a shared, centralized knowledge representation (following our design principle outlined in Section 1.2), enabling asynchronous access to the information in a way similar to how brains work.

### 2.2 Subsystems

A *Subsystem* provides a wrapper between the representations used by external components and the ones used within iCub-HRI, which compares to the façade software engineering pattern (Gamma et al., 1994). This has several advantages, including that the complexity of remote procedure calls is hidden from the user and that formerly “incompatible” components can now be used within the same project. Within this paper, we provide a brief list of the most commonly used interfaces of these subsystems, and we provide a complete list in the documentation on GitHub.^4^

This is especially evident in the subsystems for the *Actions Rendering Engine* (ARE; follow up work on Pattacini et al. (2010))^5^ and *KARMA* (Tikhanoff et al., 2015)^6^ object manipulation libraries, which are typically used by the iCub community to issue high-level motor commands. If directly called, they require the provision of complex parameters. Contrary, using iCub-HRI, one simply specifies the desired action and the name of the object to be manipulated, as further demonstrated in Section 4.1.

Other important subsystems are those for speech recognition and synthesis. Both are convenience wrappers for the functionality offered in the “speech” repository of the iCub ecosystem. The speech synthesizer allows for speech production from text using a single command “say(),” with the only parameter being the sentence to be spoken, while being agnostic to the underlying synthesizer (Acapela,^7^ eSpeak,^8^ Festival,^9^ and SVOX Pico^10^ are supported). The speech recognizer relies on the Microsoft Speech API,^11^ which allows recognition and extraction of words from spoken utterance given a grammar file (using the command “recogFromGrammarLoop()”).

The functionality of the different subsystems is aggregated in the *icubClient* class, which allows using the different subsystems from within a single class instance. A configuration file is used to specify which subsystems a module requires, such that no unnecessary resources are bound. Adding new subsystems is straightforward and we provide a tutorial to do so.^12^

## 3 iCub-HRI Modules

The modules accompanying the iCub-HRI library can be grouped into four main areas: 1. perception, 2. action, 3. social interaction, and 4. miscellaneous tools. All modules have access to the knowledge introduced in the previous section (as they use the iCub-HRI library) and none of them is required to run; i.e., one can choose which subset of modules to run for each experiment, if any.

### 3.1 Perception Modules

#### 3.1.1 Agent Detector

The *agentDetector* module is responsible for detecting and tracking a human partner using a RGB-D camera mounted behind the robot. It converts the joint positions detected by the RGB-D camera in the reference frame of the iCub and continuously updates the joint positions of the human partner in the working memory.

#### 3.1.2 Default Speech Recognition

The *Ears* module allows for recognition of speech utterances from the human when no other module is trying to recognize speech. It takes the role of a central component to redirect the command extracted from the recognized sentence to the appropriate module, while still allowing other modules to access the speech recognition subsystem directly if needed.

#### 3.1.3 Object Recognition

The object recognition module within iCub-HRI is based on the *interactive object learning* (*IOL*) pipeline (Pasquale et al., 2015). Given the two input images of the iCub’s eyes, the scene is first segmented into the background and the different objects. Each object is then classified and stereo vision (Fanello et al., 2014) is used to localize the objects. We rely on superquadric models to estimate the size and pose of objects (Vezzani et al., 2017), and we use the OpenCV object tracker (Kalal et al., 2012) to track them even if they are manipulated by the human.

#### 3.1.4 Saliency

The module *PASAR* (Mathews et al., 2012) detects the appearance and disappearance of objects, and the saliency of an object is increased proportionally to its acceleration. This also allows simple detection of pointing actions by measuring the proximity of the human’s hand with each of the objects and increasing the saliency with inverse proportion to the distance.

#### 3.1.5 Face and Action Recognition

To recognize faces and actions performed on objects, we use the *Synthetic Sensory Memory* module (Martinez-Hernandez et al., 2016). It uses Gaussian Process Latent Variable Models (Damianou et al., 2011) to train classifiers for faces and actions, which can then be loaded during interaction to perform real-time classification.

### 3.2 Action Modules

#### 3.2.1 Face Tracking

The face tracking module detects the face of a human based on Haar cascades implemented in OpenCV (Viola and Jones, 2001) and uses the velocity control of the iCub to follow the face. This module can be used in human–robot interaction scenarios for increased vividness of the robot.

#### 3.2.2 Babbling

The *babbling* module allows the issue of pseudo random (sinusoids) commands to the iCub (either individual or several joints). It has been used to learn forward and inverse models for the iCub (Zambelli and Demiris, 2017), as well as to learn correspondences between the robot’s body parts and that of the human (Zambelli et al., 2016). Within the scope of this paper, it is mainly used for body part learning, as described in Section 4.2.

### 3.3 Social Interaction Modules

#### 3.3.1 Proactive Tagging

The proactive tagging module can be used to acquire the names of objects (robot), body parts, and human partners. As this module plays a central role in the knowledge acquisition tutorial, it is further detailed in the corresponding Section 4.2.

#### 3.3.2 Reactive Layer

The reactive layer implements drive reduction mechanisms for self-regulating the robot’s behavior. A drive is defined as a control loop that triggers appropriate behaviors whenever an associated internal state variable goes out of its homeostatic range. These drives present a way to self-regulate value in a dynamic and autonomous way (Sanchez-Fibla et al., 2010). This has been shown to positively influence the acceptance of the human-robot interaction by naive users (Vouloutsi et al., 2014; Lallée and Verschure, 2015).

In the social robotic context, we provide two examples of drives that allow the robot to balance knowledge acquisition and expression in an autonomous way. The *drive for knowledge acquisition* maintains a curiosity-driven exploration of the environment by proactively requesting information from a human about the present entities (e.g., their name). The *drive for knowledge expression* regulates how the iCub expresses the acquired knowledge through synchronized speech, pointing actions and gaze. It informs the human about the robot’s current state of knowledge and thus maintains the interaction.

### 3.4 Tools

Several tools provide preprocessing functionalities for the other modules or interact with other modules of the iCub ecosystem so they can be easily used within iCub-HRI. The *guiUpdater* translates the representations of iCub-HRI to those used within the *iCubGui*. More specifically, it allows the display of location for objects and agents stored within the working memory along with certain properties, such as their color and name. The *opcPopulator* can be used to spawn new entities in simulation and control their parameters. This allows testing new functionalities in a controlled environment, without the noise encountered when using the real robot. We further provide a *touchDetector* that connects to the iCub’s artificial skin, and clusters taxels belonging to the same body part. Finally, the *referenceFrameHandler* provides functionalities similar to that of the transform library (TF; Foote, 2013), i.e., transforming a pose from one frame (e.g., that of the RGB-D camera) to another (e.g., that of the iCub root).

## 4 Using iCub-HRI

There is a variety of use cases for iCub-HRI. We first show the ease of use of iCub-HRI in a representative example related to the object manipulation subsystem. We then introduce a tutorial which demonstrates the interplay of various components in the context of human–robot interaction. Subsequently, we briefly describe how an extended version of this tutorial has been used to tackle the symbol grounding problem in the DAC-h3 framework (Moulin-Frier et al., 2017). This is followed by a description of the implications of this library for technical and non-technical users alike. Finally, we discuss the platform independence and dependencies of iCub-HRI and provide links to the documentation and repository.

### 4.1 Example Usage of the Object Manipulation Subsystems

The GitHub repository contains a range of examples, including examples of using the *KARMA* and *ARE* subsystems to manipulate objects, i.e., grasping, pushing, or pulling them, in C++, Python, and Matlab. Some examples use *yarp::sig::Vector* instances to specify the target location (important for users looking to employ iCub-HRI as a lightweight library), while others rely on the *Object* class introduced earlier (providing a seamless integration with the contained object recognition module). Listing 1 shows an example which uses the iCub-HRI library to pull an object using the *KARMA Subsystem*, while Listing 2 contains code directly communicating with *KARMA*, which is much less intuitive and likely distracts from the actually desired code related to the human–robot interaction.

**Listing 1:** Pushing an object using iCub-HRI is straightforward and requires the provision of just two parameters: the object to be pushed and the desired target position.

#include <yarp/os/all.h>
#include <icubhri/clients/icubClient.h>
int main() {
yarp::os::Network yarp;
icubhri::ICubClient iCub("KARMA_Simple");
if(!iCub.connect()) { return -1; } // connect to subsystems
std::string objectName = "octopus"; // as recognized by object recognition
double targetPositionX = -0.45;
bool ok = iCub.pushKarmaFront(objectName, targetPositionX);
yInfo() << (ok ? "Success" : "Failed");
return 0;
}

**Listing 2:** Pushing an object communicating directly with KARMA. Besides being less readable, this code is also more error prone as the Bottle’s components need to be provided with the right type and in the right order. Furthermore, many more parameters are involved.

#include <yarp/os/all.h>
#include <yarp/sig/all.h>
yarp::sig::Vector getPos(std::string name) {
// communicate with object recognition module to obtain object position
// this is not shown for brevity
}
int main() {
yarp::os::Network yarp;
yarp::os::RpcClient toKarma; toKarma.open("/example/toKarma");
yarp::os::Network::connect(toKarma.getName(), "/karmaMotor/rpc");
yarp::sig::Vector pos = getPos("octopus");
double targetPositionX = -0.45;
double radius = fabs(pos[0] - targetPositionX);
yarp::os::Bottle cmd, reply;
cmd.addString("push");
cmd.addDouble(pos[0]); cmd.addDouble(pos[1]); cmd.addDouble(pos[2]);
cmd.addDouble(-90); // angle theta
cmd.addDouble(radius); // distance to be pushed
toKarma.write(cmd, reply);
bool ok = (reply.get(0).asVocab()==yarp:os:Vocab:encode("ack"));
yInfo() << (ok ? "Success" : "Failed");
return 0;
}

### 4.2 Knowledge Acquisition Tutorial

**Figure 1:**
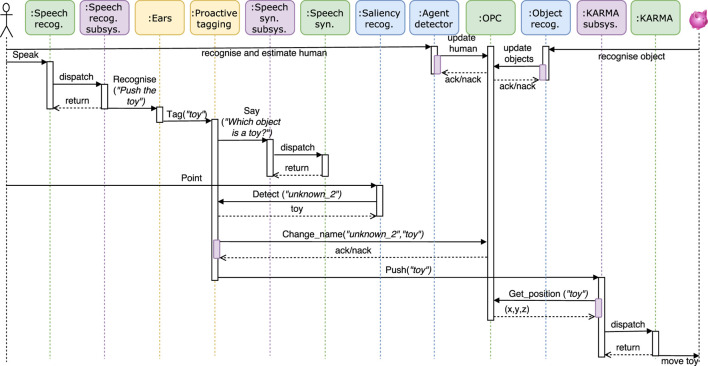
Temporal UML diagram for an interaction where a human gives a speech command to the iCub to push an object which is currently unknown to the robot. The diagram depicts the involved modules and subsystems, and shows the information flow. After converting the speech command in an action plan, the robot first asks the human to indicate the desired object, and subsequently pushes that object. The knowledge database is continuously being updated by the agent detector and object recognition system throughout the interaction, and the object name is updated after the human indicated the object by pointing to it. In our GitHub repository, we provide another diagram for the case that a drive threshold is hit, which triggers the behavior to tag an unknown object autonomously.

The robot can acquire knowledge in two different ways: proactively, where a decaying drive to acquire knowledge triggers the behavior to obtain the name of an object or body part, or reactively, where the knowledge acquisition follows a human command. The demo for this paper is centered around the proactive tagging module, which makes use of several subsystems and connects directly to several other modules. For example, it uses the speech recognition subsystem to acquire the names of entities (objects in the vicinity, partners, and body parts), the speech synthesis subsystem to enable the robot to verbally express itself (in order to ask for object names), and the *ARE* subsystem to point at objects and make them salient. Furthermore, it makes use of the functionalities provided by a number of other modules presented within the previous section, including *PASAR* to detect which object the partner is pointing to, the face recognition module to recognize the partner, and the *touchDetector* to identify which skin patch was being touched by the human. An overview of the interaction between the modules is shown in Figure 1. All further details, including the necessary set-up, configuration files, modules to run, and supported interactions, are described in the dedicated tutorial. We provide a set of videos of this experiment which demonstrates the robustness of the framework in various environments.^13^

### 4.3 Usage within DAC-h3 Framework

An extended version of the knowledge acquisition tutorial has been used to solve the symbol grounding problem, acquire language capabilities, execute goal-oriented behavior, and express a verbal narrative of the robot’s experience in the world, using the DAC-h3 framework (Moulin-Frier et al., 2017). The work of Moulin-Frier et al. (2017) also demonstrates that the software framework presented in this paper can be readily used to study human–robot interaction experiments with naive subjects.

**Figure 2:**
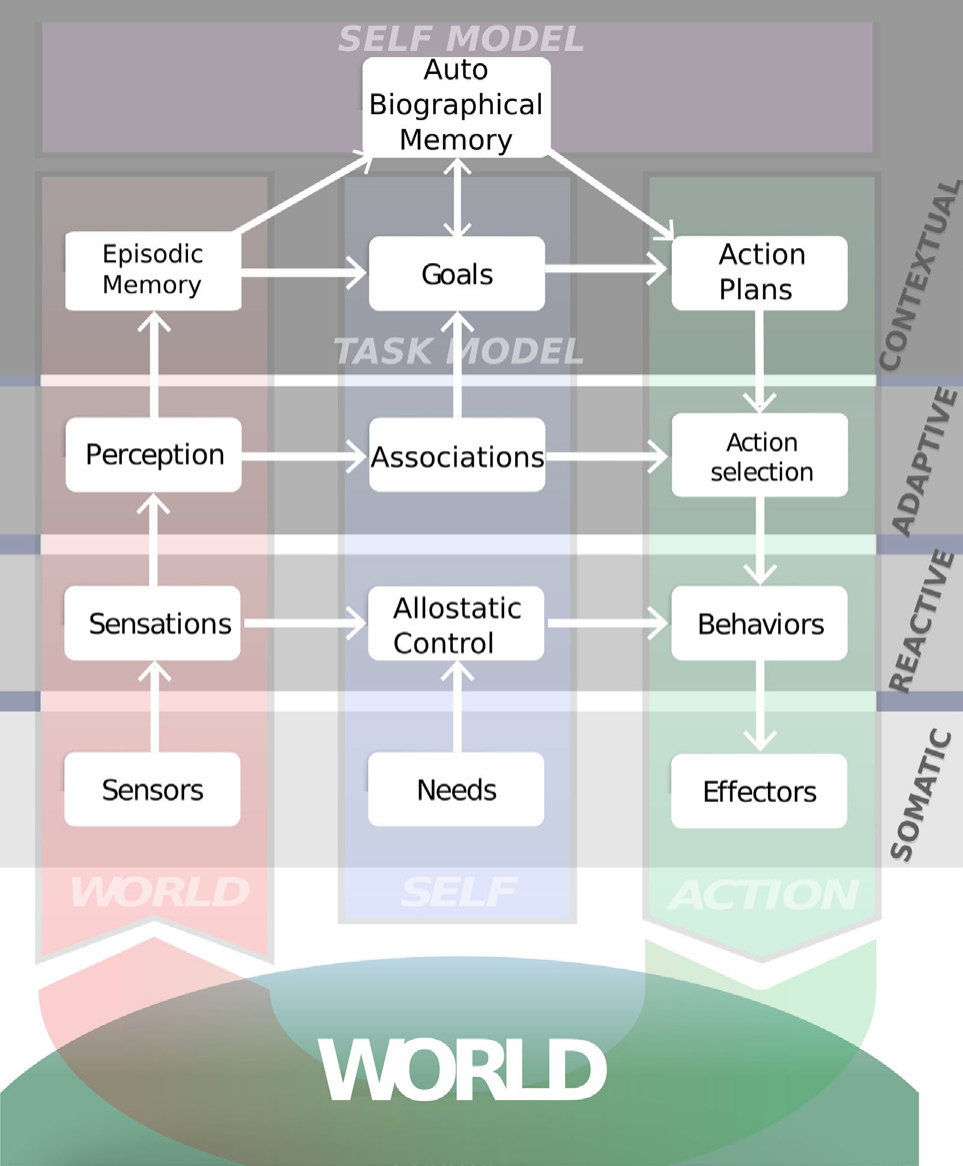
iCub-HRI serves as underlying software framework for the depicted DAC-h3 cognitive architecture (see text for more detail). The usage within DAC-h3 has shown that several design principles were successfully implemented: iCub-HRI was easy to adapt and was extended with several other modules. Furthermore, the user study presented by Moulin-Frier et al. (2017) was directly based on the knowledge acquisition tutorial presented in Section 4.2.

From the engineering perspective, the library and modules of iCub-HRI have been embedded in the Distributed Adaptive Control architecture (DAC, mentioned in the Introduction). The DAC architecture proposes that the brain can be seen as a multi-layered control structure consisting of (1) the body (with its sensors, needs and effectors), (2) the reactive layer for reflexive predefined control, (3) the adaptive layer for state acquisition and model-free reinforcement learning, and (4) the contextual layer which acquires model-based goal-oriented policies. Across these layers, we can distinguish columns of systems that processes states of the environment, the self and action as depicted in Figure 2.

The implementation of iCub-HRI with its subsystems and working memory make it particularly suitable in any scenario where module integration is driven by a complex multi-layered control architecture, with heterogeneous modules communicating within and between the different control layers.

### 4.4 More Applications and Use Cases

The central advantage of iCub-HRI is that the library bypasses the requirement for obtaining a working knowledge of the operation of a large range of modules during the normal operation of the iCub and their interaction before starting to develop one’s specific application on top of these modules. Furthermore, iCub-HRI’s modular subsystem architecture means that one can easily integrate applications developed on top of iCub-HRI to further abstract and accelerate the development of robotics applications.

The underlying design principles of iCub-HRI (see Section 1.2) and the high-level abstractions of the robot’s basic input and output systems like speech, vision, and motor control allow a wide, varied range of use cases. For users with a non-technical background, it significantly reduces the learning curve to exploit the iCub robotic platform, with potential applications such as robotic art, research into the societal effects of robotics, investigations into human–robot collaboration and human–robot interaction studies investigating the psychological effects of such an interaction. For users more familiar with the iCub, the flexibility of the library allows them to focus on the core of their applications, where iCub-HRI provides a bridge to quickly integrate these applications with the sensory, motor, and affective systems of the robot. This reduces the implementation effort which leads to faster developments, and allows for accelerated prototyping of embodied artificial intelligence applications.

### 4.5 Platform Independence

This paper specifically aims to provide a software framework to be used on the iCub humanoid robot. However, due to the modular design of the framework, certain components could be used on other robotic platforms as well, as they do not directly interface with the iCub’s sensors and/or actuators and are hence robot agnostic. The following components are platform independent and can directly be used on other robots^14^:

- Working memory (Section 2.1).
- Perception modules: agent detector (Section 3.1.1), speech recognition (Section 3.1.2), saliency (Section 3.1.4), as well as face and action recognition (Section 3.1.5).
- Reactive layer (Section 3.3.2); the actions executed by the drives can be easily adjusted in a configuration file.
- Tools (Section 3.4): the *opcPopulator* as well as the *referenceFrameHandler*.

All other components are tailored for the iCub and would need to be re-implemented or substituted with alternatives on another platform.

### 4.6 Dependencies

The only hard dependencies of iCub-HRI are a C++ 11 compatible compiler and YARP. Due to the aspiration to combine various components within a single architecture, there are a number of soft dependencies: *OpenCV, IOL*, and *superquadric-model* for object tracking, *kinect-wrapper* to track the human partner, the *speech* repository for speech synthesis and recognition, as well as (a modified version of) *KARMA* for object manipulation. All dependencies are released under free software licenses, specifically LGPLv2.1 for YARP, BSD-3-Clause in case of OpenCV and GPLv2 for all other dependencies.

The installation of these components is further detailed in the iCub-HRI repository and we provide a Python script to easily keep all dependencies up-to-date. It is also possible to download or compile a Docker image which contains all required and optional libraries pre-installed and configured.

### 4.7 Download, Licensing, and Compatibility

The code is available for download on the designated GitHub repository^15^ alongside the documentation (including class diagrams) and tutorials. It is released under the free software license GPLv2. The build status is continuously monitored on Windows, Linux, and macOS. The code itself can be considered stable and has been in adapted from the code which was used in the EFAA and WYSIWYD projects for several years.

## 5 Conclusion and Future Work

We presented iCub-HRI, a software framework which integrates various components available within the iCub ecosystem and makes them easily accessible by the means of method calls. iCub-HRI can be used in various ways, from a very lightweight library up toan integrated platform for studies on human–robot interaction. While it is tailored for the iCub humanoid robot, many parts are platform independent and can be used on other robotic platforms as well. We provide a full documentation and various tutorials, allowing researchers to easily adapt iCub-HRI for their purposes.

One limitation of the presented framework is that while it facilitates communication between different modules, it does not have any means of manipulating the execution of individual modules. This is a disadvantage in case of, e.g., monitoring real-time constraints, which cannot be guaranteed on the framework level but only within individual components (this is the case for the low-level Cartesian controller employed by ARE and KARMA (Pattacini et al., 2010)). Furthermore, as a central memory is being employed, there is a delay of the information flow from one module to another. Another limitation of this work is that no test data are being provided. Providing a proper test-suite is beyond the scope of this research, as it would need to write test cases for several tens of modules (many of them being external), and their communication handled by over 100 YARP ports. Writing suitable test cases using the testing framework presented by Natale et al. (2016) is an interesting research idea which we would like to tackle in future works.

A key point for the future adaptation of iCub-HRI will be the integration of new components from within the iCub ecosystem as well as state of the art software from related disciplines. For example, we intend to replace the current object tracking functionality with a state of the art object tracker (Choi et al., 2017); and to embed the reaching-with-avoidance framework (Nguyen et al., 2016; Roncone et al., 2016) for safer robot actions.

## Ethics Statement

The research protocol was approved by the Parc de Salut MAR—Clinical Research Ethics Committee.

## Author Contributions

TF, CM-F, DC, and J-YP drafted the initial version of the paper. TF, SL, and PN designed and implemented the iCub-HRI library. TF, J-YP, DC, PN, CM-F, and SL designed and implemented the iCub-HRI modules. TF, PN, J-YP, and SL documented the code and wrote tutorials. TF, J-YP, DC, PN, and CM-F conceived and performed the knowledge acquisition demonstration. CM-F, J-YP, and PV designed the DAC-h3 cognitive architecture. PV conceived and coordinated the EFAA and WYSIWYD projects including the proactive tagging benchmark. GM, TP, PV, and YD created the idea, were significantly involved in reviewing manuscript drafts, and supervised the project.

## Conflict of Interest Statement

The authors declare that the research was conducted in the absence of any commercial or financial relationships that could be construed as a potential conflict of interest. The reviewer, TD, declared a shared affiliation, though no other collaboration, with one of the authors, GM, to the handling editor.

## Footnotes

^1^http://efaa.upf.edu/.

^2^http://wysiwyd.upf.edu/.

^3^https://subversion.apache.org/.

^4^https://robotology.github.io/icub-hri/ → iCub-HRI libraries → Subsystems.

^5^The following interfaces are provided by the ARE subsystem: (1) “home()” to put the robot or a specified part in the home position, (2) “take()” to reach and grasp an object, (3) “push()” to laterally push an object, (4) “point()” to an object, (5) “expect()” to extend the hand and wait for an object, (6) “drop()” an object which is currently held, (7) “wave()” the robot’s hands, (8) “look()” at an object, and (9) “track()” a moving object.

^6^The following interfaces are provided by the KARMA subsystem: (1) “pushKarmaLeft()” and “pushKarmaRight()” to push an object to the left/right side with a specified target position, (2) “pushKarmaFront()” to push an object forwards, and (3) “pullKarmaBack()” to bring an object closer to the robot.

^7^http://www.acapela-group.com.

^8^http://espeak.sourceforge.net/.

^9^http://www.cstr.ed.ac.uk/projects/festival/.

^10^https://github.com/robotology/speech/tree/master/svox-speech.

^11^https://msdn.microsoft.com/en-us/library/ee125663(v=vs.85).aspx.

^12^https://robotology.github.io/icub-hri/ → Tutorials → Create a new Subsystem.

^13^https://github.com/robotology/wysiwyd—“Demonstration 4,” up to the fourth minute of the video.

^14^Provided they run on YARP, or can be interfaced with YARP through, e.g., the YARP-ROS interoperation.

^15^https://github.com/robotology/icub-hri.
